# Supplementary material for: Potassium fertilization combined with crop straw incorporation alters soil potassium fractions and availability in northwest China: An incubation study
Source: PLoS One. 2020 Jul 24;15(7):e0236634. doi: 10.1371/journal.pone.0236634 (PMC7380607; doi:10.1371/journal.pone.0236634)
Supplement: Attachment — Submitted filename: Response to Reviewers.docx [file pone.0236634.s003.docx]

**Response letter**

Dear Reviewer(s),

Thank you for your constructive and positive comments, which were valuable and helpful for revising and improving the quality of our paper. After carefully considering your comments, we revised our manuscript accordingly. We would like submit a revised version of the manuscript that addresses the points raised during the review process, and hope our revisions meet or exceed the expectations of the Editor(s) and Reviewers.

**Our point-by-point responses to the Editor(s) and Reviewers’ comments are as follows:**

1 **Ln 2 Title: should be changed, see the attached file.**

**Response:** Thank you very much for your valuable comments. We have re-phrased the title accordingly. We improved the title of article according to your comments by changing the "changes" to "improves". We think that this can well generalize our research and conclusions.

2 **Ln 22 change chemical K to mineral K, here and elsewhere.**

**Response:** We have changed the "chemical K" to "mineral K" throughout our manuscript.

3 **Ln 35 Decipher Mf and IR.**

**Response:** Thank you very much for your review of our manuscript and your valuable suggestion. We apologize for this omission. We have revised this section according to your comment, deciphered both the M_F_ and I_R_ at their first appearances.

4 **Ln 138 Can you provide an average of K content (available and/or total).**

**Response: We have added the total K data according to your suggestion. Please see in Line 139.**

5 **Ln 152 Use another symbol (e.g., K1). reader would get confused whether K refers to 200 mg K_2_O or to potassium.**

**Response:** We thanks for your valuable suggestion and apologize for this omission. We have changed the abbreviation of K fertilization from "K" to "K1" throughout our manuscript.

6 **Ln 154 What the content of K in these organic substrate. These materials will decompose differently (Wheat C:N is 80:1 while maize C/N is 60:1 as per the USDA).**

**Response:** We have provided the K concentration in wheat and maize straws according to your comments. Please see the section in **Line 149-150.**

7 **Ln 426-435 SAK was always higher after K+WS than after K+MS which may contradict the statement below. Either wheat had a higher content of K (selective absorption of K by wheat) or the urea that was added accelerated the decomposition of wheat straw given it is C rich.**

**Response:** We apologize for this lack of clarity and have improved this section according to your suggestions. The original intention of this paragraph was that compared with wheat straw (80:1 C/N ratio and 20% lignin content), maize straw (57:1 C/N ratio and 14% lignin content) is easier to decompose and promote the activity of microorganisms in the soil, thus improving the bio-chemical properties of soil. However, increased soil quality may promote the immobilization of soil available nutrients through multiple factors, including microbial utilization, aggregate occlusion, etc. The SAK is one of the sub-fractions of soil available K, and which is closely associated with soil AK. Thus, the SAK was always higher after K+WS than after K+MS.

In our research, the tested wheat straw (14.5% K content) did not have a much higher content of K than maize straw (12.5% K content), and the straws both had a total C content of about 40%. In addition, both the added urea and superphosphate to the incubated soil were equal among all treatments, which supplied available nutrients for microbial metabolism. For these, we did not think that the slight difference of K content in added straws and the equal urea input in all treatments can result in a large difference of K efficiency ratio existing between wheat and maize straw addition. In contrast, the difference in quality between wheat and maize straws seems to explain our results here.

I have good faith that the quality of the manuscript is further improved and will be ready for publication in ***PLOS ONE***. Please let me know if you have any questions.

**Best regards,**

**Xiushuang Li**
