# Supplementary material for: Potassium fertilization combined with crop straw incorporation alters soil potassium fractions and availability in northwest China: An incubation study
Source: PLoS One. 2020 Jul 24;15(7):e0236634. doi: 10.1371/journal.pone.0236634 (PMC7380607; doi:10.1371/journal.pone.0236634)
Supplement: Attachment — Submitted filename: Response to reviewers.docx [file pone.0236634.s004.docx]

**Response letter**

Dear Reviewer(s),

Thanks very much for your constructive and positive comments, which were valuable and helpful for revising and improving the quality of our paper. After carefully considering your comments, we revised our manuscript accordingly. We would like submit a revised version of the manuscript that addresses the points raised during the review process, and hope our revisions meet or exceed the expectations of the Editor(s) and Reviewers.

**Our point-by-point responses to the Editor(s) and Reviewers’ comments are as follows:**

i) the results, conclusion are mainly based on 60 d lab incubation of a 250 g soil . That is not sufficient for drawing a conclusion on decomposition of organic substrates and altering K fractions, at least for many of top expert in the field. I suggested that the title include "an incubation study” but authors did not address it. Also, the word improve in the titles does not really reflect what really happened. What does it mean to improve "soil non-specific adsorptive K" or "NEK"?does it mean to increase it or decrease it.

**Response:** We apologize for this lack of clarity and have improved this section according to your suggestions. We originally thought that although the present study was conducted using an incubation experiment, there was no need to name methods in the title, instead, it would make the title lengthy. However, after reading your comments carefully this time, we think it is really necessary to name the research method in the title in view of the limitations of this study. Thank you very much for your valuable comments. We have re-phrased the title accordingly, and changed the "improves" to "alters". We think that this can well generalize our research and conclusions.

ii) the comment on section starting Ln 417 on SAK was always higher after K+WS than after K+MS have speculations on immobilization and microbial activity that are stated as a fact. As these were not lab or field verified, it should be stated as speculation.

**Response:** Thank you very much for your review of our manuscript and your valuable suggestion. We apologize for this omission. We also think that it is necessary to indicate that it is a speculation when analyzing theories that not directly related to the present study, so as to ensure the accuracy and rigor of the study. We have revised this section according to your comment.

I have good faith that the quality of the manuscript is further improved and will be ready for publication in ***PLOS ONE***. Please let me know if you have any questions.

Best regards,

Xiushuang Li
